# Empowering Youth Amid Crises: A Call for Inclusive Child and Adolescent Public Health

**DOI:** 10.3389/phrs.2025.1607801

**Published:** 2025-03-10

**Authors:** Rick Yang, Alina Yang

**Affiliations:** ^1^ Department of Psychology, Harvard College, Cambridge, MA, United States; ^2^ Scarsdale Public Schools, Scarsdale, NY, United States

**Keywords:** adolescent health, youth, youth participation, youth participatory research, community empowerment

Dear Editors,

We read with great interest the recent commentary discussing the adverse impact of recent crises on child and adolescent health in Europe [1]. As students with extensive advocacy experience in youth mental health and cardiovascular disease, we find the proposed actions by the European Public Health Association (EUPHA) Child and Adolescent Public Health Section both timely and imperative. The piece accurately portrays the vulnerability of adolescents amidst compounded crises, from the COVID-19 pandemic to the war in Ukraine, and rightly emphasizes the need for inclusive strategies to mitigate these impacts.

The call to consult with youth populations and treat them as normal stakeholders resonates deeply with our advocacy work. Throughout our high school years, we have been actively involved in integrating youth voices into public health conversations. Such experiences and endeavors have shown us that involving young people in decision-making processes is imperative now more than ever. In an ever-increasingly digital era, silent crises magnify across social media, eroding emotional wellbeing. Our challenge is not only recognizing this subtle distortion but actively countering it with empathy, awareness, and accessible resources to foster a healthier society. Step one is engaging our most often overlooked stakeholders: the youth. Young people possess unique insights into their own needs and the challenges they face. When society treats them as equals in conversations concerning their wellbeing, policies and interventions end up becoming more tailored, effective, and sustainable.

On a similar note, the commentary’s recommendation to include children and adolescents in devising questionnaires and choosing relevant topics from their perspective is equally crucial. Through our youth lived experiences, we have previously observed that data collection instruments often fail to capture the nuanced experiences of young people. However, by engaging youth through mediums such as focus groups in the design and implementation of surveys, researchers can ensure that the data collected is of quality and reflective of their realities, which in turn empowers young people by validating their experiences and perspectives.

The entirety of “Action 5: Increase Community Empowerment and Participation” [1] aligns beautifully and precisely with our experiences and beliefs; it embodies the very ethos of our advocacy work in the United States. By increasing child and adolescent participation in public health research, policies, and actions, communities will witness positive and sustainable health outcomes. Such empowerment through diverse participation not only enhances self-efficacy and self-worth among these young people, but it also enriches an already robust evidence base with even more accurate and comprehensive data.

As young individuals, we believe this commentary presents a strikingly well-argued case for the need to enhance child and adolescent health in the face of multiple crises. By capitalizing on people-centered care, augmenting youth input-guided data collection, and empowering communities through participatory approaches, we can and must create a healthy future for all young people. It is our ultimate hope that these recommendations will be embraced and acted upon with the utmost urgency, in order to preserve, promote, and prioritize the wellbeing of young individuals in all public health efforts moving forward.
